# Characterization of a Novel Hepatitis C Virus Genotype 1 Subtype from a Patient Failing 4 Weeks of Glecaprevir-Pibrentasvir Treatment

**DOI:** 10.1128/MRA.00755-21

**Published:** 2021-10-14

**Authors:** Martin S. Pedersen, Ulrik Fahnøe, Lone W. Madsen, Peer B. Christensen, Anne Øvrehus, Jens Bukh

**Affiliations:** a Copenhagen Hepatitis C Program (CO-HEP), Department of Infectious Diseases, Copenhagen University Hospital, Hvidovre, Denmark; b CO-HEP, Department of Immunology and Microbiology, Faculty of Health and Medical Sciences, University of Copenhagen, Copenhagen, Denmark; c Department of Clinical Microbiology, Copenhagen University Hospital, Hvidovre, Denmark; d Department of Clinical Microbiology, Copenhagen University Hospital Rigshospitalet, Copenhagen, Denmark; e Department of Infectious Diseases, Odense University Hospital, Odense, Denmark; f Clinical Institute, University of Southern Denmark, Odense, Denmark; Portland State University

## Abstract

Limited information is available in relation to surveillance, genotyping, genome sequences, and treatment outcomes for rare hepatitis C virus variants. Here, we have characterized a novel subtype of major hepatitis C virus genotype 1, which was deep sequenced before and after treatment failure with 4 weeks of glecaprevir and pibrentasvir.

## ANNOUNCEMENT

Hepatitis C virus (HCV), which belongs to the genus *Hepacivirus* within the family *Flaviviridae*, causes liver cirrhosis and cancer (1). HCV is divided into 8 genotypes and >90 subtypes, and unassigned putative subtypes are still being detected, e.g., in Africa (2–4). By deep sequencing, we investigated an unknown subtype of HCV genotype 1 from a patient participating in the 4RIBC study (5) (EudraCT no. 2017-005179-21) who had been treated for 4 weeks with glecaprevir and pibrentasvir (Maviret). Treatment failed, and the patient was confirmed to be positive for the same virus at 12 weeks posttreatment. Subsequently, the patient was cured by 12 weeks of treatment with sofosbuvir, velpatasvir, and voxilaprevir (Vosevi). The viral load was 7.5 log IU/ml at baseline prior to treatment and 6.2 log IU/ml at failure, as quantified by the COBAS HCV assay (Roche). For sequencing, the RNA from the baseline sample (A106-Baseline) and the 12-week post-Maviret-treatment sample (A106-Post) was extracted from 100 μl of plasma with the TRIzol method (6). After RNA extraction, human rRNA was removed with the NEBNext rRNA depletion kit. Libraries were prepared with the NEBNext Ultra II directional RNA library preparation kit and sequenced with paired-end 150-bp reads on an Illumina NextSeq instrument (7). The human host reads (14,325,771 reads) were depleted by HISAT2 v.2.1.0 (8) mapping to the human genome hg37 (GenBank accession no. GCA_000001405.13). The unmapped reads (13,106,372 reads) were subjected to *de novo* assembly by IVA v.1.0.8 (9). Subsequent mapping and consensus calling were performed with BWA MEM and SAMtools with the single open reading frame (ORF) sequence (10). All tools were run with default parameters.

The ORF was annotated with Geneious v.10.2.3 (11) based on reference strain H77 (GenBank accession no. NC_004102) and had 9,036 bp, including the stop codon. No recombination sites were detected by RDP v.5.05 (12). The sequences of the 5′ and 3′ untranslated regions were omitted after assembly (5′ untranslated region, 207 bases; 3′ untranslated region, 101 bases). The genome coverage was ∼215,000×, and the genome had a G+C content of 58%.

**FIG 1:**
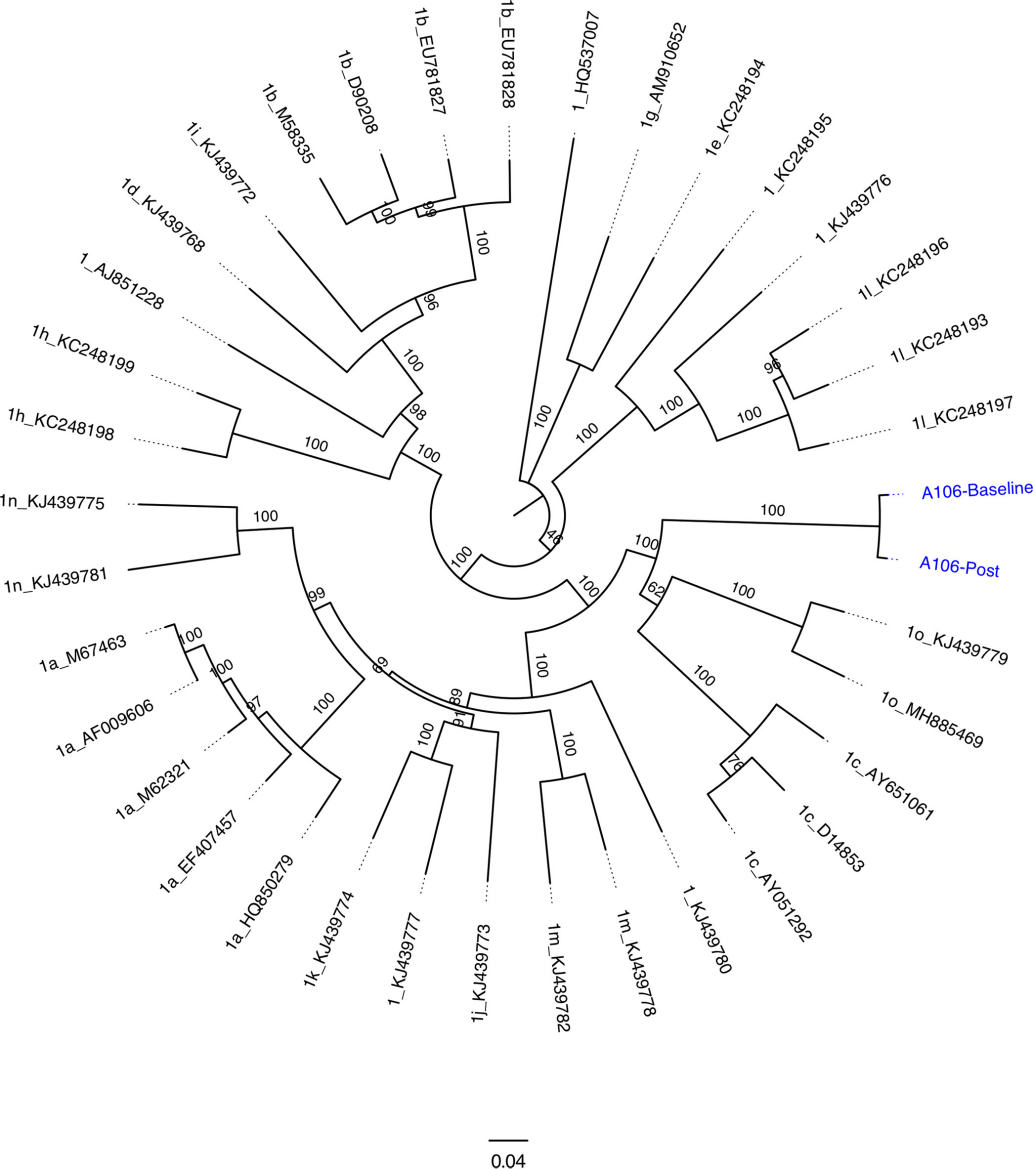
Phylogenetic tree with the genotype 1 reference sequences from the ICTV, with the two novel sequences, A106-Baseline and A106-Post, indicated in blue. The sequences were aligned with MUSCLE v.3.8.425; a maximum-likelihood phylogenetic tree was generated with IQ-TREE v.1.6.8, with 1,000 bootstrap iterations, and visualized with FigTree v.1.4.3. Each branch is labeled with the genotype number, subtype letter, and NCBI GenBank accession number. Internal branches are labeled with bootstrap support. The bar indicates substitutions per site.

The genotype 1 reference sequences were obtained from the International Committee on Taxonomy of Viruses (ICTV) (13). The genotype 1 ORF nucleotide sequences and the A106-Baseline and A106-Post sequences were aligned with MUSCLE v.3.8.425 (14), and a maximum-likelihood phylogenetic tree was created with IQ-TREE v.1.6.8, with 1,000 bootstrap iterations (15), and visualized with FigTree v.1.4.3 (http://tree.bio.ed.ac.uk/software/figtree). The pretreatment and posttreatment samples create a distinct branch from the other subtypes (Fig. 1). After treatment (A106-Post), 172 nucleotide differences (1.9%) above 50% were detected in the consensus genome by mapping and variant calling; all except 1 could be detected at baseline (A106-Baseline) as minor variants above 0.5%. Resistance-associated substitutions (RASs) were determined with HCV-GLUE v.0.1.63 (16). Before treatment initiation, multiple RASs were detected in the nonstructural protein 3 (NS3) protease region and NS5A (NS3, 56F and 170I; NS5A, 24K, 28M, 30Q, 31M, and 37L), while none was found in the NS5B polymerase region. All RASs were found in 96 to 100% of the genome population reads and remained after treatment failure, without further additions.

The closest reference strain was 1o_KJ439779 from Africa (GenBank accession no. KJ439779.1), with a consensus sequence identity of ∼80% at the nucleotide level. The viral genomes in this study originated from a patient who had immigrated from Africa to Denmark in 1996, with known potential prior exposure to HCV in Nigeria, and rare genotype 1 subtypes in Africa have been found to have lower sustained virologic response (SVR) rates (2). The genome sequence presented and the appertaining information are important for future care of patients infected with HCV subtypes that are not commonly detected.

### Data availability.

The genome sequences have been deposited in GenBank with accession no. MZ541883 and MZ541884 for A106-Baseline and A106-Post, respectively. The human read-depleted sequencing reads have been deposited in the NCBI database under BioProject no. PRJNA745515.
